# Interactive behavior in mothers with and without borderline personality disorder: non-hostile behavior is associated with stronger neural activation of the theory of mind network in response to sad faces of the own child

**DOI:** 10.3389/fpsyt.2025.1642483

**Published:** 2026-02-20

**Authors:** Kristina Meyer, Catherine Hindi Attar, Christian Banzhaf, Katja Boedeker, Ines Boegen, Katja Dittrich, Christine Heim, Sabine C. Herpertz, Charlotte Jaite, Dorothea Kluczniok, Corinne Neukel, Sina K. Poppinga, Salomé Porten, Stefan Roepke, Nikola Schoofs, Felix Bermpohl

**Affiliations:** 1 Charité–Universitätsmedizin Berlin, Corporate Member of Freie Universität Berlin and Humboldt-Universität zu Berlin, Institute of Medical Psychology, Berlin, Germany; 2 Charité – Universitätsmedizin Berlin, Corporate Member of Freie Universität Berlin and Humboldt-Universität zu Berlin, Department of Psychiatry and Neurosciences, Berlin, Germany; 3 Charité Universitätsmedizin – Berlin, corporate member of Freie Universität Berlin and Humboldt-Universität zu Berlin, Department of Child and Adolescent Psychiatry, Psychosomatics and Psychotherapy, Berlin, Germany; 4 Charité – Universitätsmedizin Berlin, Corporate Member of Freie Universität Berlin and Humboldt-Universität zu Berlin, NeuroCure Cluster of Excellence, Berlin, Germany; 5 Department of General Psychiatry, Centre for Psychosocial Medicine, Heidelberg University, Heidelberg, Germany; 6 Department of Clinical Psychology and Psychotherapy in Childhood and Adolescence, University of Hildesheim, Hildesheim, Germany; 7 German Center for Mental Health (DZPG), partner site Mannheim-Heidelberg-Ulm, Germany; 8 Oberberg Fachkliniken for Psychiatry, Psychosomatics, and Psychotherapy, Berlin and Brandenburg, Wendisch Rietz, Germany

**Keywords:** borderline personality disorder, fMRI, maternal care, parent hostility, parents with mental illness, theory of mind

## Abstract

**Objective:**

Borderline personality disorder (BPD) affects many facets of interpersonal functioning, including maternal caregiving. Deficits in theory of mind (ToM) may put mothers with BPD at risk of showing dysfunctional parenting behavior. The present study investigated the association between the ToM brain network activation and parental behavior using functional magnetic resonance imaging (fMRI).

**Method:**

In the present study conducted within the UBICA project (Understanding and Breaking the Intergenerational Cycle of Abuse), mothers with BPD (n=19) and healthy controls (HCs, n=30) completed an affect recognition task where they viewed sad, happy, and neutral faces of their own vs. unknown children during fMRI. Additionally, maternal non-hostility, the degree of maternal angry or irritable negative affect during mother-child-interactions, was assessed with the emotional availability scales.

**Results:**

Mothers with BPD compared to HCs showed lower performance in recognizing child facial expressions across emotions. Further, maternal non-hostility across groups was associated with higher activation of the ToM neural network including the temporoparietal junction (left TPJ; T = 4.52, p_FWE_ = .03; right TPJ: T = 4.44, p_FWE_ = .04) and the dorsomedial prefrontal cortex (dmPFC; T = 4.39, p_FWE_ = .05) in response to sad faces of mother’s own vs. unknown children.

**Conclusion:**

The results indicate reduced child affect recognition in mothers with BPD. Further, mothers showing stronger neural activation of the TPJ and dmPFC while seeing their own sad children were more non-hostile, pointing towards an important role of affective ToM in maternal care, which might be a viable therapeutic target in future studies.

## 1 Introduction

Borderline personality disorder (BPD) is characterized by difficulties in forming relationships, an unstable self-image, impulsivity, and identity problems (1). Close social relationships of individuals with BPD often show conflicts, alternating idealization and devaluation, and a lack of trust (2, 3). These difficulties manifest also in the family context. In families of mothers with BPD, mother-child relationships are often burdened (4, 5). For instance, mothers with BPD and their children have an increased risk of insecure or disorganized attachment styles (6). Family interactions are reported to be characterized by less family cohesion and lower structuring (7).

Dysfunctional parenting behavior may include overt and covert aggression towards the child, facially or vocally expressed anger, annoyance, or boredom, and frightening or threatening behaviors (8, 9). Such dysfunctional parenting behavior is a risk factor for adverse child outcomes. For example, mother-child interactions with mutual anger expression in toddlerhood predicted child conduct problems at school entry (10). In a meta-analysis, Khaleque (11) linked parental hostility/aggression, a subscale of the child version of the parental acceptance-rejection questionnaire, to child maladjustment, e.g., child aggression and emotional instability. Many BPD patients were exposed to childhood maltreatment (12), which parents with BPD describe as a strong source of motivation to do their best to create a better developmental environment for their own children (13). However, despite the parents’ best efforts, parental personality disorders, including BPD, remain a risk factor for dysfunctional parenting behavior (14). This underlines the urgent need for research to understand the intergenerational transmission pathways of dysfunctional parenting and, ultimately, improve targeted care for parents with BPD and their children (5).

Different BPD symptoms may affect parent-child interactions in parents with BPD (13, 15). Part of the social interaction difficulties in BPD may be attributed to social cognition deficits (16–18). Social cognition refers to cognitive abilities relevant to social interactions (19). It includes theory of mind (ToM), the ability to internally represent the feelings, intentions, and actions of others (20). Affective ToM refers to the representation of the feelings of others while cognitive ToM refers to the interpretation of others’ thoughts and intentions. In the context of parenthood, social cognitive abilities have been capitalized as important, allowing parents to understand their children’s needs and feelings (21, 22).

Experimental studies linked BPD symptoms to social cognition deficits. For example, Ortega-Díaz et al. (23) asked individuals with BPD, their first-degree relatives, and a sample of healthy controls (HCs) to complete tasks of different aspects of ToM. They found altered ToM processing in BPD patients relative to HCs. In a meta-analysis, Nemeth and colleagues (16) reported consistent deficits in cognitive ToM and task-dependent deficits in affective ToM in individuals with BPD.

Using functional magnetic resonance imaging (fMRI), ToM abilities have been mapped to activation in regions of the brain such as the temporoparietal junction (TPJ), the dorsomedial prefrontal cortex (dmPFC), the posterior cingulate cortex (PCC), and the precuneus (24, 25). Previous fMRI studies have demonstrated increased neural responses of brain regions associated with ToM as well as empathy upon seeing children in social situations in mothers compared to age-matched non-mothers, hinting towards the relevance of social cognition in motherhood (26, 27).

FMRI studies have shown that the brain regions activated upon seeing one’s own as opposed to an unknown child diverge (28), indicating specific neural processes that allow mothers to engage in social interactions with their children. Because the risk of parent-child interaction problems is higher in demanding emotional situations, and because sadness is thought to be particularly difficult for many BPD patients (29, 30), we focus on neural responses to children’s sad faces.

Given the role of ToM in successful parenting, we will focus on the ToM brain network (24, 31) in mothers with BPD. We use data from the multicentric UBICA project (Understanding and Breaking the Intergenerational Cycle of Abuse) on maternal parenting behavior observed in mother-child interactions, performance in an affect recognition task with own and unknown sad child faces, and the ToM network activation in response to these faces. In a previous study from the UBICA project, Kluczniok et al. (32) found that mothers with BPD showed less non-hostile parenting behavior as assessed using the emotional availability (EA) scales (8, 9) than mothers with remitted major depression and HCs. While the present study uses partly the same behavioral data, we focus here on a sub-sample who participated in the fMRI study.

In the present study, we examined whether dysfunctional parenting behavior, specifically the display of maternal irritability, anger, or aggression, is associated with altered affective ToM at the behavioral and neural level. At the behavioral level, we hypothesize that 1) mothers with BPD show impaired affect recognition, which we deem a precursor ability related to affective ToM, and that 2) the affect recognition performance for sad child faces is associated with maternal dysfunctional parenting behavior. At the neural level, we hypothesize that 3) mothers with BPD activate their ToM network less strongly than HCs when seeing faces of their own versus unknown sad children, and that 4) the ToM network activation in own vs. unknown sad children is related to the extent of maternal dysfunctional parenting behavior.

## 2 Methods

### 2.1 Sample description

19 mothers with BPD and 30 HC mothers (**Table 1**) participated in the study that was approved by the ethics committee of the Charité – Universitätsmedizin Berlin (approved 27.08.2013, EA2/097/13). All 49 participated in the fMRI study and had sufficient data quality after fMRI quality control (**Supplementary Material 1** contains quality control criteria). One mother in the BPD group and one in the HC group did not participate in the mother-child interaction task due to time constraints unrelated to the study design, reducing sample sizes in these sub-analyses by one per group.

**Table 1:** Sample characteristics and demographic and clinical variables.

Variable name	BPD (n = 19)		HCs (n = 30)		*t*	*p*
	*M*	*SD*	*M*	*SD*		
Age (mother)	34.8	4.8	39.8	5.4	3.3	0.002
BDI-II	24.6	15.2	4.3	5.1	-6.6	<.001
PSI	141	34	171.8	36.4	2.8	0.004
Child sex: female	47.40%		56.70%			
History of abuse assessed using CECA:						
Neglect: none	4 (21.1%)		25 (83.3%)			
Neglect: low	4 (21.1%)		5 (16.7%)			
Neglect: moderate	6 (31.6%)		0			
Neglect: severe	4 (21.1%)		0			
Emotional abuse: none	10 (52.6%)		30 (100%)			
Emotional abuse: low	2 (10.5%)		0			
Emotional abuse: moderate	3 (15.8%)		0			
Emotional abuse: severe	4 (21.1%)		0			
Physical abuse: none	2 (10.5%)		23 (76.7%)			
Physical abuse: low	7 (36.8%)		7 (23.3%)			
Physical abuse: moderate	7 (36.8%)		0			
Physical abuse: severe	3 (15.8%)		0			
Sexual abuse: none	6 (31.6%)		27 (90.0%)			
Sexual abuse: low	3 (15.8%)		2 (6.7%)			
Sexual abuse: moderate	2 (10.5%)		0			
Sexual abuse: severe	8 (42.1%)		1 (3.3%)			
Reported medication						
Antidepressant	3 (15.8%)		0			
Antiepileptics	1 (5.2%)		0			
Thyroid	1 (5.2%)		4 (13.3%)			
Other	1 (5.2%)		3 (10%)			

BPD, mothers with borderline personality disorder; HCs, healthy controls; BDI-II, Beck depression inventory (33); PSI, parental stress index (34); CECA, child experiences of care and abuse interview (35).

Mothers from both groups and their biological children aged 5–12 years (M = 8.31, SD = 1.97) were required to live together and be proficient in German. In the BPD group, inclusion criteria were a current BPD diagnosis (per the International Personality Disorder Examination interview, IPDE) (36) and remission from mood episodes as per a score of < 8 in the Hamilton Depression Scale (37, 38).

In the HC group, mothers were included if they reported no current or lifetime axis I or II disorders in the IPDE or the Mini International Neuropsychiatric Interview (MINI) (39).

Mothers were excluded in case of: acute suicidality, being in an emotional crisis or hospitalized, neurological diseases, current alcohol or drug dependence, a history of schizophrenia or manic episodes per the MINI, anxious-avoidant or antisocial personality disorder per the IPDE, use of benzodiazepines within the past six months (40), change of psychotropic medication two weeks before study entry.

Children were excluded if they had a diagnosis of autistic disorder (based on DSM-IV criteria) or an IQ score below 70 in the Culture Fair Intelligence Tests (41).

In the BPD group, current and lifetime comorbid disorders were assessed using the MINI (39). Of the mothers with BPD, n = 17 reported a history of previous depressive disorders, n = 4 reported current comorbid anxiety disorders, n = 1 a current obsessive compulsive disorder, n = 3 reported current substance abuse, and n = 2 reported a current eating disorder. N = 5 mothers with BPD reported no history of psychiatric hospitalizations, n = 4 reported one hospitalization, n = 10 reported two or more hospitalizations.

### 2.2 Data collection

#### 2.2.1 Maternal non-hostility assessed using the emotional availability scales

The EA scales are an observation tool for parent-child interactions that yields the parental scales non-hostility, non-intrusiveness, sensitivity, and structuring (9). The validity of the EA scales is suggested, for instance, by convergent validity with other parent-child interaction observation tools (42) and criterion validity through associations with parent and child attachment and parental mental illness (43, 44) as reviewed in (8).

Because Kluczniok, Boedeker (32) reported less non-hostile parenting behavior, but unaffected sensitivity in mothers with BPD, we focus on maternal non-hostility for the present analysis.

In the first 15 minutes of the video-recorded parent-child interaction, dyads played with a standardized set of age-appropriate toys as they normally would. After the free play, the children were given a puzzle (Shape by Shape) at a difficulty level slightly too high for their age for 6 minutes. Mothers were instructed to assist the child without solving the puzzle for them.

Three trained raters evaluated maternal non-hostility on a scale of 1 to 7 with higher scores signifying lack of hostility, higher scores signifying covert hostility (e.g., subtle signs of annoyance or boredom, slightly raising one’s voice, responding irritably), and lowest scores signifying overtly aggressive or threatening behaviors. All videos were coded by at least two raters blind to participant diagnosis. Discrepancies were resolved through discussion. Inter rater reliability ranged between r = .84 and r = .90 for the non-hostility scale, indicating good to excellent agreement (45).

#### 2.2.2 MRI task

The task was developed for the purpose of the UBICA project (46, 47) and heeded common recommendations for timing and stimulus presentation in fMRI (48). In the scanner, mothers completed an affect recognition task in a 3x2 design (facial emotion: happy, sad, neutral; child identity: own child vs. unknown child) with child face stimuli generated for the present study. Individual child images of the participants’ children were created beforehand in a separate mood induction session [**Supplementary Material 2** and (46)]. During fMRI, a total of 180 images were presented in a pseudo-randomized order. 50% of the images showed the own child, the other 50% the unfamiliar child. A trial started with the presentation of a child face (2s) followed by a fixation cross for a randomly chosen period of 2-6s. Mothers were instructed to classify the affective facial expression as quickly and accurately as possible by button press using the index (randomized for sad/happy), middle (for neutral) and ring finger (randomized for sad/happy) of their right hand. The task was distributed across two runs of 8 minutes each.

#### 2.2.3 MRI scanning parameters

MRI scanning parameters are described in **Supplementary Material 3**.

### 2.3 Data analysis

#### 2.3.1 Behavioral data

Behavioral data were analyzed using R (49) and different packages (50–57). We conducted a 2x3x2 analysis of variance (ANOVA) to investigate the effect of group (BPD vs. HC), child facial expression (sad, neutral, happy), and child identity (own child vs. unknown child) on response times (RTs) and hit rates of the within-scanner affect recognition task. Greenhouse-Geisser corrections were applied whenever deemed necessary by sphericity tests (58). Further, bivariate Pearson correlations between performance in recognizing sad faces and maternal non-hostility were computed.

We performed one-sided significance tests and applied Bonferroni corrections to the ANOVAs’ *post-hoc* pairwise comparisons and Pearson correlations.

#### 2.3.2 MRI data

Data analysis of the imaging data was conducted using the statistical parametric mapping (SPM12) in MATLAB. Preprocessing is described in **Supplementary Material 4**.

At the first level, neural responses were modelled using a general linear model including six regressors representing all combinations of child identity (own, unknown) and facial emotion (sad, happy, neutral).

To test group differences in neural activation to sad expressions of one’s own versus an unfamiliar child, the first-level contrast (sad own > sad unknown) was computed for each participant. These individual contrast images were then entered into a two-sample t-test to compare BPD and HC groups (HC > BPD and BPD > HC), with age included as a covariate.

We tested for an association between maternal behavior and brain activation by regressing non-hostility on ToM network activation while viewing sad faces of the own vs. unknown children in SPM12. For this, groups were pooled. Maternal age was entered as a nuisance regressor to the model.

All hypothesis-driven second-level analyses of brain activation using fMRI – group comparisons and regressions – were restricted to an *a priori* ToM network mask. This region of interest (ROI) included the bilateral TPJ, dmPFC, PCC, and precuneus, based on the predefined ToM template from (24). The use of an a priori–defined template of the ToM network was chosen to facilitate comparability with previous research on ToM network modulations in clinical samples (59). Small-volume correction within this mask was applied (FWE p <.05). Using a single combined ToM mask ensured a conservative and theory-guided testing.

To validate the findings derived from this template-based ROI, we additionally employed a functional ROI of the regions significantly (whole-brain analysis, p < 0.01 uncorrected) activated in the own sad child > unknown sad child contrast generated by a one-sample t-test in the current sample (**Supplementary Material 5**).

To explore potential effects outside the ToM network, we additionally conducted whole-brain analyses at a threshold of FWE-corrected p <.001 (**Supplementary Material 6**).

## 3 Results

### 3.1 Behavioral results: maternal non-hostility

Mothers with and without BPD showed overall mostly non-hostile parenting behavior with average ratings between five and six, indicating mostly covert signs of anger, impatience, or boredom (e.g., slightly raising one’s voice, rolling eyes, snapping at the child or responding irritably), but the HCs were rated significantly more non-hostile than mothers with BPD (MHCs = 5.93 ± 0.96; t = 2.6, p = .01; MBPD = 5.08 ± 1.31). This was in line with the results from the total UBICA sample reported in Kluczniok et al. (32).

### 3.2 Behavioral results, hypothesis 1: group differences in affect recognition

**Figure 1:**
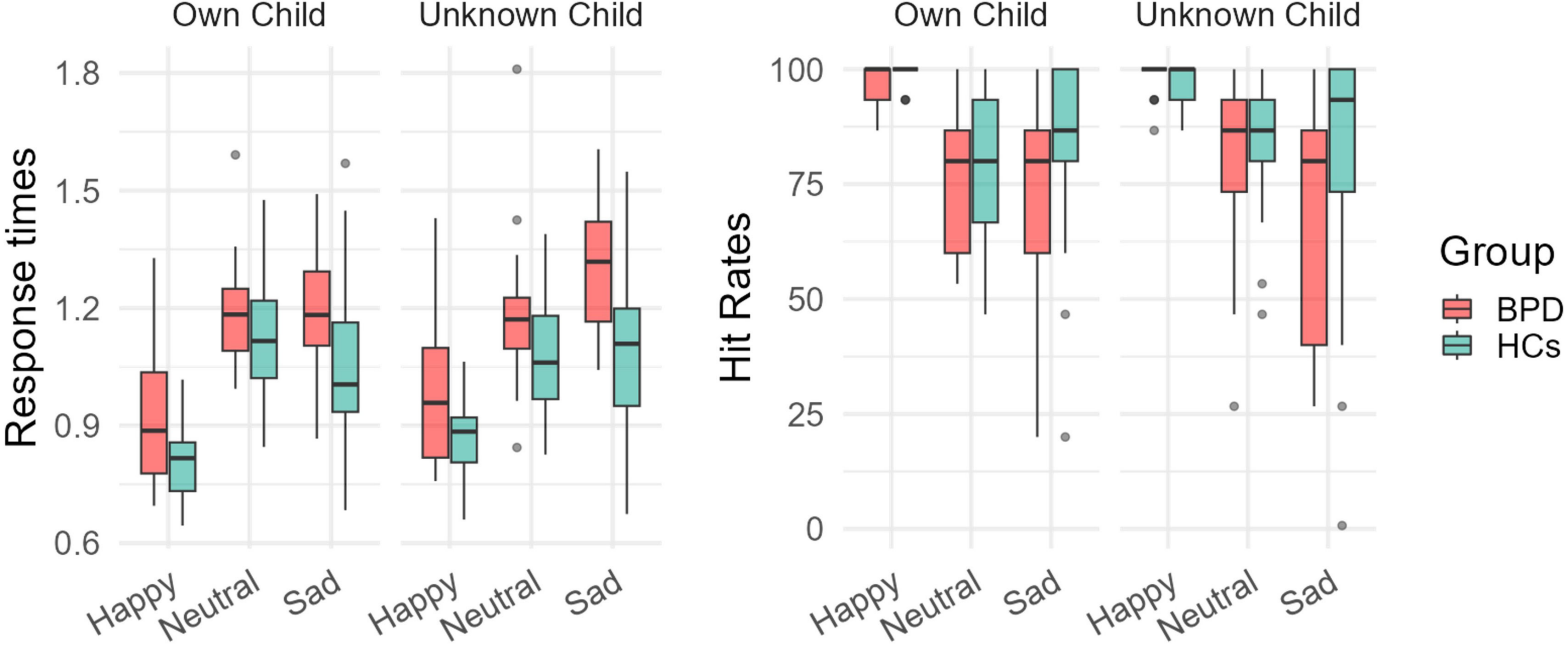
Boxplots of response times in seconds and hit rates in % correct responses in the inside-scanner affect recognition task. The task followed a 3x2 design with emotion (happy, neutral, sad) and identity (own child vs. unknown child) as within-subject factors. BPD, mothers with borderline personality disorder. HCs, healthy control mothers. The black crossbars represent mean values, the boxes represent the 25^th^ to 75^th^ quartile, the whiskers represent values up to 1.5 times the interquartile range outside the 25^th^ and 75^th^ quartiles.

In the following, ANOVA results for RTs and hit rates of the affect recognition task are reported (**Figure 1**, **Table 2**). In two individuals of the BPD and one of the HC groups, hit rates were 0% in at least one condition. In a sensitivity analysis, we excluded these individuals and repeated the ANOVA analyses, but the results remained identical in interpretation. Therefore, we report the results with the full sample.

**Table 2:** Descriptive statistics of performance in the inside-scanner affect recognition task.

Group	Emotion	Identity	Hit rates (%)		Response time (s)	
			M	SD	M	SD
BPD	Happy	Own Child	97.89	3.88	0.92	0.18
BPD	Happy	Unknown Child	97.89	3.88	0.98	0.18
BPD	Neutral	Own Child	73.68	23.46	1.19	0.15
BPD	Neutral	Unknown Child	77.19	26.13	1.17	0.22
BPD	Sad	Own Child	64.91	32.80	1.21	0.16
BPD	Sad	Unknown Child	61.75	32.95	1.28	0.18
HCs	Happy	Own Child	99.31	2.07	0.82	0.10
HCs	Happy	Unknown Child	97.93	3.61	0.88	0.09
HCs	Neutral	Own Child	78.39	17.06	1.13	0.16
HCs	Neutral	Unknown Child	84.37	13.72	1.07	0.14
HCs	Sad	Own Child	85.29	19.30	1.05	0.21
HCs	Sad	Unknown Child	81.40	24.82	1.09	0.20

BPD, mothers with borderline personality disorder; HCs, healthy control mothers.

#### 3.2.1 Response times

There were significant main effects of group (F _(1, 44)_ = 14.7, p <.001), emotion (F_(2, 88)_ = 64.8, p <.001), identity (F_(1, 44)_ = 5.2, p = .03), and a significant emotion*identity interaction (F_(2, 88)_ = 4.9, p = .02). Pairwise comparisons revealed that mothers with BPD compared to HCs showed longer response times (p <.001), confirming Hypothesis 1. Faces of own children were generally processed faster than faces of unknown children (p = .03). Happy faces were processed faster than neutral and sad faces (p <.001), but there was no difference between sad and neutral faces (p = 1.0).

The emotion*identity interaction indicated that the processing of own child faces was faster than unknown child faces for sad (p = .03) and happy (p <.001) facial expressions, but there was no difference between the speed of processing neutral own vs. unknown child faces (p = .24).

#### 3.2.2 Hit rates

There were significant main effects of group (F_(1, 46)_ = 10.6, p = .002), emotion (F_(2,80)_ = 35.7, p <.001), and a significant group*emotion interaction (F_(2, 80)_ = 5.1, p = .01). Pairwise comparisons indicated that HCs compared to BPD showed higher hit rates (p = .002), confirming Hypothesis 1. Further, happy faces were identified with higher accuracy compared to neutral (p <.001) and sad (p <.001) facial expressions, but there was no significant difference between neutral and sad faces (p = .5).

The group*emotion interaction revealed that the main effect of group was driven by the lower hit rate in sad faces in the BPD vs. HC group (p = .002), whereas there were no differences between mothers with BPD and HCs in happy (p = .36) and neutral (p = .23) faces.

### 3.3 Behavioral results, hypothesis 2: association between affect recognition and non-hostility

None of the correlations between hit rates or RTs in the affect recognition task of sad faces with maternal non-hostility were significant after correcting for multiple comparisons (*p* values >.16), disconfirming Hypothesis 2.

### 3.4 FMRI results, hypothesis 3: group differences in ToM network activation when seeing own vs. unknown sad children

For the HC > BPD contrast of own > unknown sad child faces, we observed a non-significant (ROI p_FWE_ = .28) activation within the left dmPFC (x = -12, y = 50, z = 30; t = 3.6), disconfirming Hypothesis 3. In the reversed BPD > HC contrast of own > unknown sad children, no group differences in ToM network activation emerged.

### 3.5 fMRI results, Hypothesis 4: association between ToM network activation and maternal non-hostility

**Figure 2:**
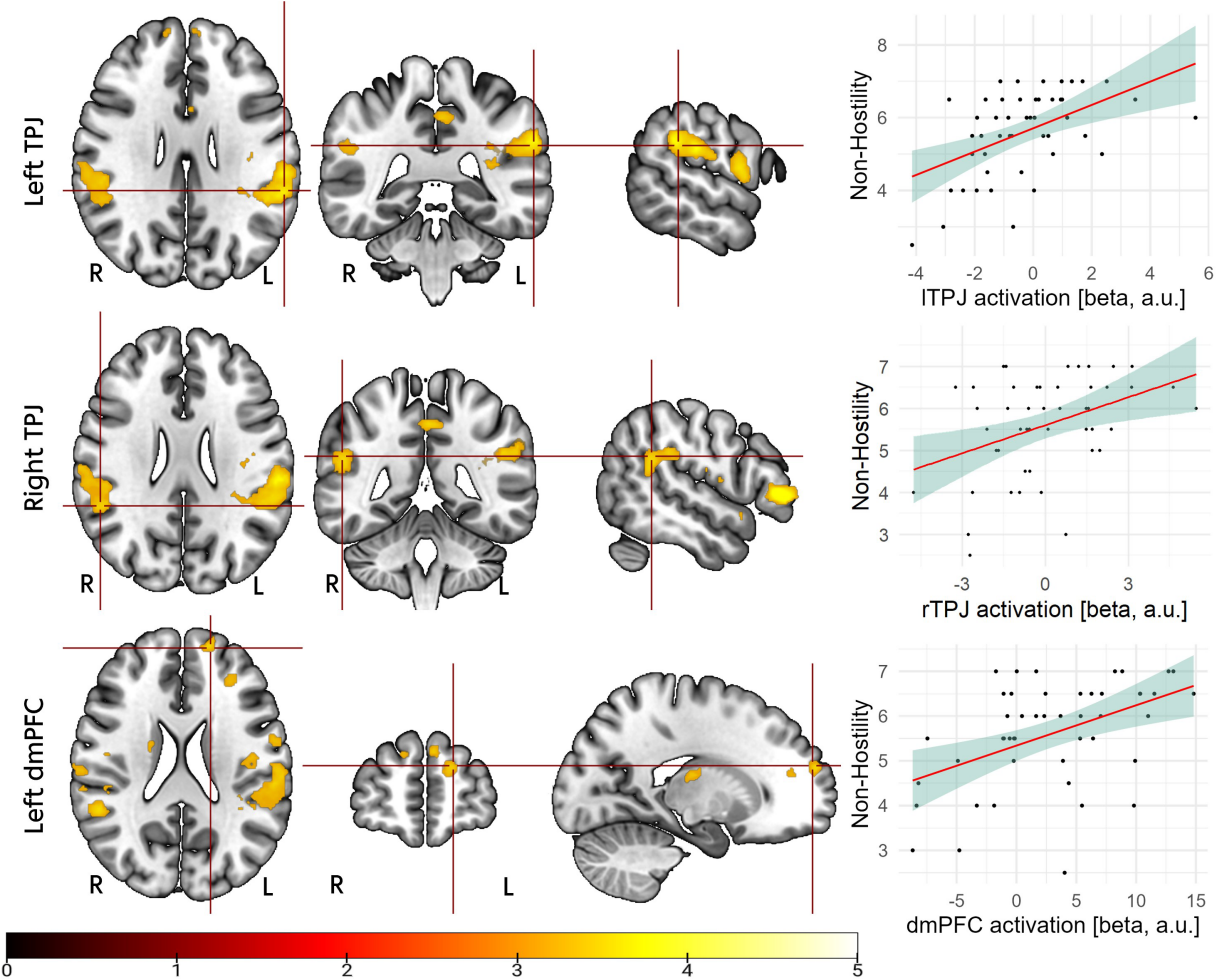
Upper part: activation in the left temporoparietal junction (lTPJ; x = -50, y = -44, z = 28) associated with maternal non-hostility, measured with the emotional availability scales (60); middle part: activation in the right temporoparietal junction (rTPJ; x = -50, y = -44, z = 28) associated with maternal non-hostility; lower part: activation in the left dorsomedial prefrontal cortex (dmPFC; x = -16, y = 58, z = 20) associated with maternal non-hostility. For illustration purposes, masks of the activated clusters were used and activations are overlaid on the Montreal Neurological Institute (MNI152) template embedded in MRIcroGL software (61). For illustration purposes only, beta values were exported from the mean activations of the respective activated cluster and correlated off-line with non-hostility ratings.

In mothers with and without BPD, associations between higher non-hostility scores and brain activation while viewing sad faces of their own vs. unknown children were significant for the left TPJ (x = -50, y = -44, z = 28; T = 4.52; ROI p_FWE_ = .03), right TPJ (x = 52, y = -44, z = 22; T = 4.44; ROI p_FWE_ = .04), and left dmPFC (x = -16, y = 58, z = 20; T = 4.39, ROI p_FWE_ = .05), confirming Hypothesis 4 (**Figure 2**).

To explore if the association between ToM network activation and maternal behavior was driven by differences in positive parenting behavior, we regressed maternal sensitivity during the mother-child interaction on ToM network activation but found no significant relationship (T < 3.69, ROI p_FWE_ >.23).

There were no activations significantly associated with maternal non-hostility in the precuneus or PCC.

## 4 Discussion

Mothers with borderline personality disorder (BPD) and healthy control (HC) mothers in our sample showed overall high scores of non-hostility falling in the range of covert, subtle signs of irritability, anger, or boredom (8, 9), but HCs were rated more non-hostile than mothers with BPD.

Our hypothesis that mothers with BPD vs. HCs would show longer response times and lower accuracies in the inside-scanner affect recognition task with sad, neutral, and happy faces of their own vs. unknown children, was confirmed by our data, indicating impaired child affect recognition in BPD. For hit rates, an interaction between group and emotion indicated that the effect was mainly driven by lower performance for sad faces in mothers with BPD compared to HCs, whereas happy and neutral faces were recognized with equal accuracy across groups. In the pooled group of mothers with BPD and HCs, consistent with our hypotheses, maternal non-hostility was positively associated with activation in the bilateral temporoparietal junction (TPJ) and dorsomedial prefrontal cortex (dmPFC), key regions of the ToM network, when viewing sad faces of their own versus unknown children.

Contrary to our hypotheses, we found no significant association between maternal ToM ability in the affect recognition task with sad faces and maternal non-hostility, and no significant group differences between mothers with BPD and HCs in ToM brain network activation while seeing sad faces of own vs. unknown children.

### 4.1 Behavioral findings

Mothers with BPD showed a reduced ability to understand children’s facially expressed emotions. Stepp et al. (62) suggest that mothers with BPD have difficulties understanding and regulating their own and their children’s emotions, possibly leading to higher ambiguity in emotional social interactions. Problems in dealing with other individuals’ emotional expressions is further implied by studies showing that individuals with BPD have difficulties in understanding the feelings of others (63) and find emotionally loaded social situations more overwhelming than HCs (64). The findings of the present study support this view. Possibly, in social interactions with their children, mothers with BPD find it harder than HCs to understand their child’s feelings, causing higher ambiguity and possibly, higher parenting stress (65). As indicated by an interaction between group and emotion showing difficulties of mothers with BPD in identifying sad faces, this might be most prevalent in situations where their children are sad.

However, we found no association between affect recognition performance and maternal non-hostility. This absence of a direct correlation suggests that the basic ability to recognize a sad face may be too distant from the complex demands required for displaying non-hostile parenting behavior during real-life interaction. In a study by Kiel et al. (66), mothers with BPD showed the same negative affect towards their child’s distress as HCs in the strange situations test but were less likely to engage in positive soothing behavior. Therefore, the difference may lie not in emotion recognition itself but in the downstream expression, regulation, or utilization of emotions to maintain positive behavior.

Future studies should employ more faceted, naturalistic affective ToM tasks. Further, studying mother-child social interactions might benefit from employing more emotionally salient stimuli such as infant and child crying, as these are expected to elicit stronger maternal emotional responses (67, 68).

### 4.2 Neural correlates of maternal non-hostility

Our results suggest that a neural correlate of non-hostile parenting behavior is increased activation of the mothers’ ToM network when viewing their own sad children. This association was found specifically for maternal non-hostility, not maternal sensitivity, highlighting the role of ToM processes, specifically affective ToM, in reducing dysfunctional parenting behavior. The regions found to be associated with non-hostility, the TPJ and dmPFC, have previously been shown to be associated with social cognition in close relationships (69). TPJ activation was, for example, shown to be positively related to self-reported parental empathy, meaning empathy with the participants’ children versus empathy in general (70). A dismissive attachment style was associated with lower TPJ activation during the *reading the mind in the eyes* test, an affect recognition task often implicated in affective ToM (71). Wever et al. (72) found the TPJ and dmPFC to be associated with empathy with the participants’ own versus unfamiliar children. The dmPFC is thought to play an important role in mentalizing processes (73), possibly enabling individuals to represent the intentions of others (74).

Together with the previous literature, our results suggest that mothers who respond in a non-hostile manner when interacting with their children show stronger activation of the TPJ and dmPFC when seeing their child displaying sadness. The role of these brain regions in maternal care is suggested (75). Further, this is compatible with the notion that interpersonal problems in mother-child relationships of mothers with BPD are partly explainable by maternal affective ToM.

We found no significant group differences between mothers with BPD and HCs in ToM network activation while seeing the sad faces of own vs. unknown children. Activation of the ToM network might be related to BPD symptoms in ways not covered by a group contrast. For example, when comparing brain activation upon seeing their own children in mothers with early life maltreatment versus non-maltreated mothers, Neukel et al. (47) reported heightened activation in ToM-related regions of the brain. Boehme et al. (76) found increased social anxiety to be related with increased TPJ and dmPFC activation in self-referential thinking. Beeney et al. (77) also reported greater activation in BPD compared to HCs in the TPJ, dmPFC, precuneus, and cingulate cortex when participants were asked to evaluate themselves. Possibly, in the present study, different symptom constellations in our BPD patients might have led to different, counteracting alterations in the ToM network activation. Alternatively, the absence of a group difference might be attributable to the small sample size, as group analyses are especially prone to power issues (78).

### 4.3 Clinical implications

The association between ToM network activation upon seeing own sad children and maternal non-hostility implies affective ToM as a potential intervention target in parents at risk for dysfunctional parenting behavior. Interventions targeting social abilities are being developed for parents and their families (79). For example, Volkert et al. (80) present a parent training directed at severely mentally ill parents that constitutes an adaptation of the lighthouse parent training against childhood maltreatment (81) interlaced with mentalization-based psychotherapy (82). Mentalization is conceptualized as the ability to represent the mental states of oneself and others and understanding current feelings and behaviors as an expression of these mental states (82).

Previous studies demonstrate that by psychotherapeutic interventions, specific neural processes can be strengthened (31, 83). For instance, in patients with bipolar disorder, Meyer et al. (31) demonstrated that a psychotherapeutic intervention with a practical focus on social functioning and perspective-taking increased the neural activation in the ToM network post-therapy. Pre- and post-intervention fMRI studies are needed to better understand the role of the ToM brain network in improving parent-child interactions.

### 4.4 Strengths and limitations

The major limitation of the present study is its small sample size, especially in the BPD group, which renders the results preliminary and limits the generalizability of the findings. Further, it is possible that fluctuating mood states in the mothers with BPD influenced the association between ToM network activation and maternal non-hostility. Repeated measurements are warranted in future research, as they would allow for stronger conclusions regarding causality, which the current cross-sectional data do not permit.

The strengths of the study lie in its integration of different data sources (observational, experimental, neuroimaging) and the use of stimuli based on images of the participants’ own children. Pooling BPD and HC groups for the regression had the advantages of increasing the variance in maternal non-hostility observed in interactions and improving the power over studying the groups separately but prevented us from detecting group-specific effects.

As described in **Table 1**, the BPD sample of the current study reported low intake of medication relative to many other patients with BPD (84), a possible consequence of the psychotherapeutic as opposed to pharmacological treatment approach of our clinic, where many of the patients were recruited, and of the exclusion of patients with current depression scores above a clinical cut-off from participation. This low intake of medication has the advantage of reducing the effect of the pharmacotherapy on brain activation.

### 4.5 Conclusion

Mothers with BPD showed difficulties recognizing facial emotion expressions compared to HCs. Maternal non-hostile behavior was related to higher bilateral TPJ and left dmPFC activation, indicating that mothers who showed more non-hostility while playing with their children employed their ToM network more strongly when seeing their own sad children. This finding underscores the relevance of affective ToM in parenting behavior and may suggest that targeting affective ToM therapeutically could benefit parent-child interaction quality.

## Data Availability

The raw data supporting the conclusions of this article will be made available by the authors, without undue reservation.

## References

[1] American Psychiatric Association . Diagnostic and statistical manual of mental disorders: DSM-5™ (5th ed.). Arlington, VA: American Psychiatric Publishing, Inc. (2013). doi: 10.1176/appi.books.9780890425596

[2] Lis S Bohus M . Social interaction in borderline personality disorder. Curr Psychiatry Rep. (2013) 15:1–7. doi: 10.1007/s11920-012-0338-z, PMID: 23307561

[3] Weinbrecht A Niedeggen M Roepke S Renneberg B . Feeling excluded no matter what? Bias in the processing of social participation in borderline personality disorder. NeuroImage: Clinical. (2018) 19:343–50. doi: 10.1016/j.nicl.2018.04.031, PMID: 30013917 PMC6044182

[4] Petfield L Startup H Droscher H Cartwright-Hatton S . Parenting in mothers with borderline personality disorder and impact on child outcomes. BMJ Ment Health. (2015) 18:67–75. doi: 10.1136/eb-2015-102163, PMID: 26205740 PMC11234925

[5] Florange JG Herpertz SC . Parenting in patients with borderline personality disorder, sequelae for the offspring and approaches to treatment and prevention. Curr Psychiatry Rep. (2019) 21:1–8. doi: 10.1007/s11920-019-0996-1, PMID: 30729325

[6] Macfie J Swan SA . Representations of the caregiver–child relationship and of the self, and emotion regulation in the narratives of young children whose mothers have borderline personality disorder. Dev psychopathology. (2009) 21:993–1011. doi: 10.1017/S0954579409000534, PMID: 19583894 PMC2825084

[7] Feldman RB Zelkowitz P Weiss M Vogel J Heyman M Paris J . A comparison of the families of mothers with borderline and nonborderline personality disorders. Compr Psychiatry. (1995) 36:157–63. doi: 10.1016/S0010-440X(95)90110-8, PMID: 7758301

[8] Biringen Z Derscheid D Vliegen N Closson L Easterbrooks MA . Emotional availability (EA): Theoretical background, empirical research using the EA Scales, and clinical applications. Dev review. (2014) 34:114–67. doi: 10.1016/j.dr.2014.01.002

[9] Biringen Z . The Emotional Availability (EA) Scales. 4th Edition, Middle Childhood/Youth Version. Box PO , editor. Boulder, Colorado (2008). p. 3625.

[10] Lorber MF Egeland B . Parenting and infant difficulty: Testing a mutual exacerbation hypothesis to predict early onset conduct problems. Child Dev. (2011) 82:2006–20. doi: 10.1111/j.1467-8624.2011.01652.x, PMID: 22026438

[11] Khaleque A . Perceived parental hostility and aggression, and children’s psychological maladjustment, and negative personality dispositions: A meta-analysis. J Child Family Stud. (2017) 26:977–88. doi: 10.1007/s10826-016-0637-9

[12] Simon JJ Spiegler K Coulibaly K Stopyra MA Friederich H-C Gruber O . Beyond diagnosis: symptom patterns across complex PTSD and borderline personality disorder. Front Psychiatry. (2025) 16. doi: 10.3389/fpsyt.2025.1668821, PMID: 41244875 PMC12612632

[13] Kasiviswanathan K Lee J Rao S Broadbear JH . Navigating parenthood in people living with borderline personality disorder: a meta-ethnography. Borderline Pers Disord Emotion Dysregulation. (2025) 12:22. doi: 10.1186/s40479-025-00291-6, PMID: 40462227 PMC12131352

[14] Steele KR Townsend ML Grenyer BF . Parenting and personality disorder: An overview and meta-synthesis of systematic reviews. PloS One. (2019) 14:e0223038. doi: 10.1371/journal.pone.0223038, PMID: 31574104 PMC6772038

[15] Vanwoerden S Greiner I Ensink K Sharp C . The relations between self- and caregiver- focused reflective function and theory of mind in the context of borderline pathology in adolescence. Psychiatry Res. (2019) 273:274–80. doi: 10.1016/j.psychres.2019.01.042, PMID: 30677714

[16] Nemeth N Matrai P Hegyi P Czeh B Czopf L Hussain A . Theory of mind disturbances in borderline personality disorder: A meta-analysis. Psychiatry Res. (2018) 270:143–53. doi: 10.1016/j.psychres.2018.08.049, PMID: 30248485

[17] Galvez-Merlin A Lopez-Villatoro JM de la Higuera-Gonzalez P de la Torre-Luque A Reneses-Prieto B Diaz-Marsa M . Social cognition deficits in borderline personality disorder: Clinical relevance. Psychiatry Res. (2024) 331:115675. doi: 10.1016/j.psychres.2023.115675, PMID: 38134528

[18] Doell KC Olié E Courtet P Corradi-Dell'Acqua C Perroud N Schwartz S . Atypical processing of social anticipation and feedback in borderline personality disorder. NeuroImage: Clinical. (2020) 25:102126. doi: 10.1016/j.nicl.2019.102126, PMID: 31884223 PMC6938803

[19] Happé F Cook JL Bird G . The structure of social cognition: in(ter)dependence of sociocognitive processes. Annu Rev Psychol. (2017) 68:243–67. doi: 10.1146/annurev-psych-010416-044046, PMID: 27687121

[20] Kanske P Böckler A Trautwein F-M Singer T . Dissecting the social brain: Introducing the EmpaToM to reveal distinct neural networks and brain–behavior relations for empathy and Theory of Mind. Neuroimage. (2015) 122:6–19. doi: 10.1016/j.neuroimage.2015.07.082, PMID: 26254589

[21] Rigby J Conroy S Miele-Norton M Pawlby S Happé F . Theory of mind as a predictor of maternal sensitivity in women with severe mental illness. psychol Med. (2016) 46:1853–63. doi: 10.1017/S0033291716000337, PMID: 26979486

[22] van't Hof SR Straathof M Spalek K Hoekzema E . Theory of mind during pregnancy and postpartum: A systematic review. J neuroendocrinology. (2023) 35:e13266. doi: 10.1111/jne.13266, PMID: 37094082

[23] Ortega-Díaz E García-Campos J Moya-Martínez A Ramírez-Cremades C Rico-Gomis JM Cuesta-Moreno C . Theory of mind in borderline personality disorder: a possible endophenotypic factor? Int J Environ Res Public Health. (2021) 18:3193. doi: 10.3390/ijerph18063193, PMID: 33808735 PMC8003401

[24] Mohnke S Erk S Schnell K Romanczuk-Seiferth N Schmierer P Romund L . Theory of mind network activity is altered in subjects with familial liability for schizophrenia. Soc Cognit Affect Neurosci. (2016) 11:299–307. doi: 10.1093/scan/nsv111, PMID: 26341902 PMC4733339

[25] Schurz M Tholen MG Perner J Mars RB Sallet J . Specifying the brain anatomy underlying temporo-parietal junction activations for theory of mind: A review using probabilistic atlases from different imaging modalities. Hum Brain Mapp. (2017) 38:4788–805. doi: 10.1002/hbm.23675, PMID: 28608647 PMC6867045

[26] Plank IS Hindi Attar C Kunas SL Dziobek I Bermpohl F . Increased activation in the bilateral anterior insulae in response to others in pain in mothers compared to non-mothers. Sci Rep. (2021) 11:22757. doi: 10.1038/s41598-021-02162-w, PMID: 34815443 PMC8610985

[27] Plank IS Hindi Attar C Kunas SL Dziobek I Bermpohl F . Motherhood and theory of mind: increased activation in the posterior cingulate cortex and insulae. Soc Cogn Affect Neurosci. (2021) 17(5):470–81. doi: 10.31219/osf.io/2cxks, PMID: 34592763 PMC9071419

[28] Rigo P Kim P Esposito G Putnick DL Venuti P Bornstein MH . Specific maternal brain responses to their own child’s face: An fMRI meta-analysis. Dev Review. (2019) 51:58–69. doi: 10.1016/j.dr.2018.12.001, PMID: 30872887 PMC6411077

[29] Dixon-Gordon KL Peters JR Fertuck EA Yen S . Emotional processes in borderline personality disorder: An update for clinical practice. J Psychother integration. (2017) 27:425. doi: 10.1037/int0000044, PMID: 29527105 PMC5842953

[30] Hepp J Lane SP Carpenter RW Niedtfeld I Brown WC Trull TJ . Interpersonal problems and negative affect in borderline personality and depressive disorders in daily life. Clin psychol science. (2017) 5:470–84. doi: 10.1177/2167702616677312, PMID: 28529826 PMC5436804

[31] Meyer K Catherine HA Fiebig J Stamm T Bassett TR Bauer M . Boosting the ToM network: Specific psychotherapy increases neural correlates of affective theory of mind in euthymic bipolar disorder. Biol Psychiatry: Cogn Neurosci Neuroimaging. (2022) 8(5):572–80. doi: 10.31234/osf.io/vk5r4, PMID: 36087699

[32] Kluczniok D Boedeker K Attar CH Jaite C Bierbaum A-L Fuehrer D . Emotional availability in mothers with borderline personality disorder and mothers with remitted major depression is differently associated with psychopathology among school-aged children. J Affect Disord. (2018) 231:63–73. doi: 10.1016/j.jad.2018.02.001, PMID: 29453011

[33] Beck AT Steer RA Brown G . BDI-II: Beck Depression Inventory Manual. 2nd ed. San Antonio: Psychological Corporation; (1996).

[34] Tröster H . Eltern-Belastungs-Inventar: EBI; deutsche Version des Parenting Stress Index (PSI) von RR Abidin. Göttingen: Hogrefe (2011).

[35] Kaess M Parzer P Mattern M Resch F Bifulco A Brunner R . Childhood experiences of care and abuse (CECA). Z für Kinder-und Jugendpsychiatrie und Psychotherapie. (2011) 39(4):243–52. doi: 10.1024/1422-4917/a000115, PMID: 21667449

[36] Loranger AW Janca A Sartorius N . Assessment and diagnosis of personality disorders: The ICD-10 internationalpersonality disorder examination (IPDE). Cambridge (Camb): Cambridge University Press (1995)39(4):243–52. [WHO IRIS: apps.who.int/iris/handle/10665/41912]

[37] Hamilton M . The Hamilton Rating Scale for Depression. In: Sartorius N. Ban T. A. (eds) Assessment of Depression. Springer, Berlin, Heidelberg. (1986). doi: 10.1136/jnnp.23.1.56, PMID:

[38] Hamilton M . The Hamilton rating scale for depression. Assess depression: Springer;. (1986), 143–52. doi: 10.1007/978-3-642-70486-4_14

[39] Sheehan DV Lecrubier Y Sheehan KH Amorim P Janavs J Weiller E . The Mini-International Neuropsychiatric Interview (MINI): the development and validation of a structured diagnostic psychiatric interview for DSM-IV and ICD-10. J Clin Psychiatry. (1998) 59:22–33., PMID: 9881538

[40] Casper T Arbour MW . Identification of the pregnant woman who is using drugs: implications for perinatal and neonatal care. J Midwifery Women's Health. (2013) 58:697–701. doi: 10.1111/jmwh.12087, PMID: 24015821

[41] Cattell RB Weiß R Osterland J . Grundintelligenztest Skala 1: CFT 1. (1997) Göttingen: Hogrefe.

[42] Derscheid DJ Fogg LF Julion W Johnson ME Tucker S Delaney KR . Emotional availability scale among three US race/ethnic groups. Western J Nurs Res. (2019) 41:409–30. doi: 10.1177/0193945918776617, PMID: 29781393

[43] Ann Easterbrooks M Biringen Z . Guest editors’ introduction to the special issue: Mapping the terrain of emotional availability and attachment. Attachment Hum Dev. (2000) 2:123–9. doi: 10.1080/14616730050085518, PMID: 11707906

[44] Easterbrooks MA Bureau J-F Lyons-Ruth K . Developmental correlates and predictors of emotional availability in mother–child interaction: A longitudinal study from infancy to middle childhood. Dev psychopathology. (2012) 24:65–78. doi: 10.1017/S0954579411000666, PMID: 22292994

[45] Cicchetti DV . Guidelines, criteria, and rules of thumb for evaluating normed and standardized assessment instruments in psychology. psychol assessment. (1994) 6:284. doi: 10.1037/1040-3590.6.4.284

[46] Kluczniok D Hindi Attar C Stein J Poppinga S Fydrich T Jaite C . Dissociating maternal responses to sad and happy facial expressions of their own child: An fMRI study. PloS One. (2017) 12:e0182476. doi: 10.1371/journal.pone.0182476, PMID: 28806742 PMC5555664

[47] Neukel C Herpertz SC Hinid-Attar C Zietlow A-L Fuchs A Moehler E . Neural processing of the own child’s facial emotions in mothers with a history of early life maltreatment. Eur Arch Psychiatry Clin Neurosci. (2019) 269:171–81. doi: 10.1007/s00406-018-0929-8, PMID: 30056560

[48] Birn RM Cox RW Bandettini PA . Detection versus estimation in event-related fMRI: choosing the optimal stimulus timing. Neuroimage. (2002) 15:252–64. doi: 10.1006/nimg.2001.0964, PMID: 11771993

[49] R Core Team . R: the R project for statistical computing. 2019 (2020). Available online at: https://wwwr-projectorg/ (Accessed March 30, 2020).

[50] Wickham H . ggplot2: Elegant Graphics for Data Analysis. Springer-Verlag. (2016) New York.

[51] Wickham H François R Henry L Müller K Vaughan D . dplyr: A Grammar of Data Manipulation.(2025) 1(1):1–1.Available online at: https://dplyr.tidyverse.org.

[52] Mangiafico S . Rcompanion: Functions to Support Extension Education Program Evaluation. (2023) 2(4):1–1. Rutgers Cooperative Extension.

[53] Wickham H Bryan J . Read Excel Files. (2025)1(4):1–1. Available onlineat: https://github.com/tidyverse/readxl, https://readxl.tidyverse.org.

[54] Ram K Wickham H . wesanderson: A Wes Anderson Palette Generator.(2023) 0(3):7–7. Available online at: https://github.com/karthik/wesanderson.

[55] Ooms J Denney B . writexl: Export Data Frames to Excel ‘xlsx’Format. (2025) 1(5):4–4. Available online at: https://github.com/ropensci/writexl.

[56] Lenth R Lenth MR . Least-Squares Means: The R Package lsmeans. J Stat Softw. 69(1):1–33. doi: 10.18637/jss.v069.i01

[57] Singmann H Bolker B Westfall J Aust F Ben-Shachar M . afex: Analysis of Factorial Experiments.(2025) 1(5):1–1. Available online at: https://CRAN.R-project.org/package=afex.

[58] Kassambara A . rstatix: pipe-friendly framework for basic statistical tests. (2023). doi: 10.32614/CRAN.package.rstatix

[59] Hindi Attar C Ridder N Stein J Kluczniok D Dittrich K Jaite C . Maladaptive mother–child interactions in mothers with remitted major depression are associated with blunted amygdala responses to child affective facial expressions. psychol Med. (2025) 55:e15. doi: 10.1017/S0033291724003404, PMID: 39905759 PMC11968120

[60] Biringen Z Robinson JL Emde RN . Appendix B: The emotional availability scales (; an abridged infancy/early childhood version). Attachment Hum Dev. (2000) 2:256–70. doi: 10.1080/14616730050085626, PMID: 11707915

[61] Rorden C Brett M . Stereotaxic display of brain lesions. Behav neurology. (2000) 12:191–200. doi: 10.1155/2000/421719, PMID: 11568431

[62] Stepp SD Whalen DJ Pilkonis PA Hipwell AE Levine MD . Children of mothers with borderline personality disorder: identifying parenting behaviors as potential targets for intervention. Pers Disorders: Theory Research Treat. (2012) 3:76. doi: 10.1037/a0023081, PMID: 22299065 PMC3268672

[63] Domes G Schulze L Herpertz SC . Emotion recognition in borderline personality disorder—A review of the literature. J Pers Disord. (2009) 23:6–19. doi: 10.1521/pedi.2009.23.1.6, PMID: 19267658

[64] Dixon-Gordon KL Fitzpatrick S Haliczer LA . Emotion regulation and borderline personality features in daily life: The role of social context. J Affect Disord. (2021) 282:677–85. doi: 10.1016/j.jad.2020.12.125, PMID: 33445091

[65] Bonfig J Herpertz SC Schneider I . Altered hormonal patterns in borderline personality disorder mother-child interactions. Psychoneuroendocrinology. (2022) 143:105822. doi: 10.1016/j.psyneuen.2022.105822, PMID: 35709662

[66] Kiel EJ Gratz KL Moore SA Latzman RD Tull MT . The impact of borderline personality pathology on mothers' responses to infant distress. J Family Psychol. (2011) 25:907. doi: 10.1037/a0025474, PMID: 21928886

[67] Geerling I Roberts RM Sved Williams A . Impact of infant crying on mothers with a diagnosis of borderline personality disorder: A qualitative study. Infant Ment Health J. (2019) 40:405–21. doi: 10.1002/imhj.21776, PMID: 30964954

[68] Schneider I Herpertz SC Ueltzhöffer K Neukel C . Stress and reward in the maternal brain of mothers with borderline personality disorder: a script-based fMRI study. Eur Arch Psychiatry Clin Neurosci. (2024) 274:117–27. doi: 10.1007/s00406-023-01634-6, PMID: 37354380 PMC10786970

[69] Saxe R Kanwisher N . People thinking about thinking people: the role of the temporo-parietal junction in “theory of mind. Soc neuroscience: Psychol Press;. (2013) p:171–82. doi: 10.1016/S1053-8119(03)00230-1, PMID: 12948738

[70] Ojha A Miller JG King LS Davis EG Humphreys KL Gotlib IH . Empathy for others versus for one's child: Associations with mothers’ brain activation during a social cognitive task and with their toddlers’ functioning. Dev psychobiology. (2022) 64:e22313. doi: 10.1002/dev.22313, PMID: 36282757 PMC9608359

[71] Baskak B Kır Y Sedes N Kuşman A Türk EG Baran Z . Attachment style predicts cortical activity in temporoparietal junction (TPJ). J Psychophysiol. (2019) 34(2):99. doi: 10.1027/0269-8803/a000240, PMID: 33840879 PMC8034263

[72] Wever MC van Houtum LA Janssen LH Will G-J Tollenaar MS Elzinga BM . Neural signatures of parental empathic responses to imagined suffering of their adolescent child. NeuroImage. (2021) 232:117886. doi: 10.1016/j.neuroimage.2021.117886, PMID: 33617996

[73] Amodio DM Frith CD . Meeting of minds: the medial frontal cortex and social cognition. Nat Rev Neurosci. (2006) 7:268–77. doi: 10.1038/nrn1884, PMID: 16552413

[74] Moessnang C Otto K Bilek E Schäfer A Baumeister S Hohmann S . Differential responses of the dorsomedial prefrontal cortex and right posterior superior temporal sulcus to spontaneous mentalizing. Hum Brain Mapping. (2017) 38:3791–803. doi: 10.1002/hbm.23626, PMID: 28556306 PMC6866721

[75] Feldman R . The adaptive human parental brain: implications for children's social development. Trends neurosciences. (2015) 38:387–99. doi: 10.1016/j.tins.2015.04.004, PMID: 25956962

[76] Boehme S Miltner WH Straube T . Neural correlates of self-focused attention in social anxiety. Soc Cogn Affect Neurosci. (2015) 10:856–62. doi: 10.1093/scan/nsu128, PMID: 25326038 PMC4448029

[77] Beeney JE Hallquist MN Ellison WD Levy KN . Self–other disturbance in borderline personality disorder: Neural, self-report, and performance-based evidence. Pers Disorders: Theory Research Treat. (2016) 7:28. doi: 10.1037/per0000127, PMID: 26011577 PMC4659768

[78] Charness G Gneezy U Kuhn MA . Experimental methods: Between-subject and within-subject design. J economic Behav organization. (2012) 81:1–8. doi: 10.1016/j.jebo.2011.08.009

[79] Rosenbach C Heinrichs N Kumsta R Schneider S Renneberg B . Study protocol for a multi-center RCT testing a group-based parenting intervention tailored to mothers with borderline personality disorder against a waiting control group (ProChild*-SP1). TRIALS. (2022) 23:589. doi: 10.1186/s13063-022-06531-2, PMID: 35870944 PMC9308114

[80] Volkert J Georg A Hauschild S Herpertz SC Neukel C Byrne G . Strengthening Attachment Competencies in Parents with Mental Illness: Adaptation and Pilot Testing of the Mentalization-Based Lighthouse Parenting Program/Bindungskompetenzen psychisch kranker Eltern starken: Adaptation und Pilottestung des mentalisierungsbasierten Leuchtturm-Elternprogramms. Praxis der Kinderpsychologie und Kinderpsychiatrie. (2019) 68:27–43. doi: 10.13109/prkk.2019.68.1.27, PMID: 30628875

[81] Byrne G Sleed M Midgley N Fearon P Mein C Bateman A . Lighthouse Parenting Programme: Description and pilot evaluation of mentalization-based treatment to address child maltreatment. Clin Child Psychol Psychiatry. (2019) 24:680–93. doi: 10.1177/1359104518807741, PMID: 30387373

[82] Bateman AW Fonagy P . Mentalization-based treatment of BPD. J Pers Disord. (2004) 18:36–51. doi: 10.1521/pedi.18.1.36.32772, PMID: 15061343

[83] Meyer K Attar CH Fiebig J Stamm T Bassett TR Bauer M . Daring to feel: emotion-focused psychotherapy increases amygdala activation and connectivity in euthymic bipolar disorder—A randomized controlled trial. Biol Psychiatry: Cogn Neurosci Neuroimaging. (2023) 8:750–9. doi: 10.1016/j.bpsc.2023.02.008, PMID: 36898634

[84] Bridler R Häberle A Müller ST Cattapan K Grohmann R Toto S . Psychopharmacological treatment of 2195 in-patients with borderline personality disorder: a comparison with other psychiatric disorders. Eur Neuropsychopharmacol. (2015) 25:763–72. doi: 10.1016/j.euroneuro.2015.03.017, PMID: 25907249

