# Supplementary material for: Interactive behavior in mothers with and without borderline personality disorder: non-hostile behavior is associated with stronger neural activation of the theory of mind network in response to sad faces of the own child
Source: Front Psychiatry. 2026 Feb 20;16:1642483. doi: 10.3389/fpsyt.2025.1642483 (PMC12963725; doi:10.3389/fpsyt.2025.1642483)
Supplement: Supplementary file 1 [file SupplementaryFile1.docx]

**Supplement 1: Quality control criteria for functional magnetic resonance imaging (fMRI) data**

The following quality control steps were conducted: 1) visual inspection of raw EPI images to identify artifacts (signal dropout, ghosting, excessive noise), 2) visual inspection of the realignment plots to ensure that images were within the motion parameter thresholds (< 3 mm translation and < 3° rotation), 3) visual inspection after normalization by overlaying individual normalized images on the DARTEL template.

After applying motion thresholds, the remaining datasets showed low and stable head motion, with no abrupt spikes indicative of excessive framewise displacement. Each run included 250 volumes (TR = 2 s; total acquisition time ≈ 8 minutes). Given this relatively short duration, additional denoising (e.g., FD–based scrubbing) was not applied, as it would have reduced usable volumes and risked compromising model estimation and statistical power. Residual motion or physiological artifacts may therefore remain, representing an inherent limitation of short fMRI runs.

**Supplement 2: Mood induction for generating child images**

Individual child images of the participants’ children were created beforehand in a separate mood induction session described also in (1). Specifically, children were first video-taped while looking in a relaxed way into the camera to obtain neutral faces, then asked to remember a sad situation they experienced recently and to behave (i.e. walk, look) as if they were sad again. This was followed by video recordings of the children while they first watched short sad video sequences (e.g. “The Lion King” by Disney) to obtain sad faces and then short funny video sequences (e.g. “Mickey Mouse” by Disney) for happy faces. From the videos, images were created, transformed to grey scale and rated by an independent trained rater team for emotional valence on a 7-point Likerst-scale ranging form very sad (-3) to very happy (+3) with a final selection of 30 images for each valence rated at least moderate (+/-2) for emotional, and always 0 for neutral images.

Control stimuli of unfamiliar children were created using the same procedure as for the experimental stimuli. Specifically, we collected images of six children (three boys and three girls), aged six, eight, and ten years, each displaying happy, neutral, and sad facial expressions. For each participant, we then selected the control stimulus set that best matched the experimental stimuli in both age and gender. All images were converted to grayscale and normalized for mean luminance using the same image-processing pipeline. This procedure ensured that the stimuli were matched in basic visual properties and that any observed neural effects could not be attributed to differences in color or brightness. Images were cropped in a way that only the faces were visible. Faces of own and unfamiliar children were not matched in terms of physical similarity (e.g., eye colour, face shape).

**Supplement 3: Scanning parameters**

MRI data were acquired using a 3 Tesla Siemens Trio whole-body scanner (Siemens Healthcare, Erlangen, Germany) equipped with a 32-channel head coil. Functional images were collected in two runs, each consisting of 250 volumes, with a voxel size of 3 × 3 × 3 mm³. Each volume comprised 33 axial slices. High-resolution structural images were acquired using a T1-weighted MPRAGE sequence (192 slices; voxel size = 1 × 1 × 1 mm³). Functional images were T2*-weighted and obtained using an echo-planar imaging (EPI) sequence with a repetition time (TR) of 2000 ms, echo time (TE) of 30 ms, flip angle of 78°, field of view (FOV) of 192 × 192 mm², and an in-plane matrix of 64 × 64.

**Supplement 4: MRI data preprocessing**

Functional images were preprocessed using SPM12 (Wellcome Trust Centre for Neuroimaging, London, UK). For each of the two scanning sessions of the affect recognition task, images underwent slice-timing correction and were realigned to the first volume using “Realign (Estimate & Unwarp),” which estimates six rigid-body motion parameters and corrects for susceptibility-by-motion interactions. The mean functional image was coregistered to the participant’s structural T1-weighted image. Structural images were segmented into gray matter, white matter, and cerebrospinal fluid using SPM12 tissue probability maps, and individual DARTEL flow fields were calculated with existing CAT12 templates. Functional images were normalized to MNI space via the DARTEL flow fields. We smoothed the data using an 8-mm FWHM Gaussian kernel in line with the procedure applied in another recent study from our group using the same scanning protocol (2) as well as common heuristics recommending a smoothing kernel of about 2-3 times the voxel size (3, 4). Finally, a high-pass filter of 128 s was applied, and serial correlations were modelled using an AR(1) process.

**Supplement 5: Functional ROI results based on the sample data**

Based on the one-sample t-test for the contrast own sad child > unknown sad child across both groups, three significant ToM regions were identified (whole-brain analysis, p < 0.01 uncorrected):

- **PCC/Precuneus:**  −6 −52 22; T = 6.43; p = 0.001
- **Right dmPFC:**  10 54 40; T = 5.99; p = 0.005
- **Right TPJ:**  54 −56 14; T = 5.29; p = 0.039

Using these three regions combined into a single mask and small-volume correction within this mask (FWE p < .05) for the group comparison HC > BPD, we observed a nonsignificant dmPFC activation, consistent with the ROI analysis reported in the main manuscript (left dmPFC: −12 48 32; T = 3.57; p = 0.314).

Across mothers with and without BPD, higher non-hostility scores were associated with greater brain activation when viewing sad faces of their own versus unknown children, with a significant effect in the left dmPFC ( −16 58 20; T = 4.39; p = 0.047) and a trend-level effect in the right TPJ (52 −46 18; T = 4.31; p = 0.058).

**Supplement 6: Exploratory whole-brain analysis of MRI data**

In all whole-brain analyses, clusters activated above a significance threshold of family-wise error (FWE) corrected p < .001 are reported. In all whole-brain analyses, the first-level contrast sad own child > sad unknown child is investigated. In two-sample t-tests controlling for age as a covariate of no interest, this yielded no significant activations. In the regression analysis of brain activation on maternal non-hostility, the exploratory whole-brain analysis yielded a cluster in the right inferior frontal gyrus that was positively associated with maternal non-hostility (T = 6.41, p_FWE_ = .002, x = 50, y = 36, z = 2).

**Supplement references**

1. Kluczniok D, Hindi Attar C, Stein J, Poppinga S, Fydrich T, Jaite C, et al. Dissociating maternal responses to sad and happy facial expressions of their own child: An fMRI study. PloS one. 2017;12(8):e0182476.

2. Hindi Attar C, Ridder N, Stein J, Kluczniok D, Dittrich K, Jaite C, et al. Maladaptive mother–child interactions in mothers with remitted major depression are associated with blunted amygdala responses to child affective facial expressions. Psychological Medicine. 2025;55:e15.

3. Poldrack RA, Mumford JA, Nichols TE. Handbook of functional MRI data analysis. New York: Cambridge University Press; 2011.

4. Chen Z, Calhoun V. Effect of Spatial Smoothing on Task fMRI ICA and Functional Connectivity. Frontiers in Neuroscience. 2018;Volume 12 - 2018.
